# Effect of Direct Oral Anticoagulant, Patient, and Surgery Characteristics on Clinical Outcomes in the Perioperative Anticoagulation Use for Surgery Evaluation Study

**DOI:** 10.1055/s-0040-1716512

**Published:** 2020-09-23

**Authors:** Kira MacDougall, James D. Douketis, Na Li, Nathan P. Clark, Alfonso Tafur, Julien D'Astous, Joanne Duncan, Sam Schulman, Alex C. Spyropoulos

**Affiliations:** 1 Department of Medicine, Northwell Health, Staten Island University Hospital, New York, New York, United States; 2 Department of Medicine, Thrombosis and Atherosclerosis Research Institute, McMaster University, Hamilton, Ontario, Canada; 3 Kaiser Permanente Colorado, Aurora, Colorado, United States; 4 Northshore University Health System, University of Chicago, Pritzker School of Medicine, Evanston, Illinois, United States; 5 University of Ottawa, Ottawa, Ontario, Canada; 6 Zucker School of Medicine at Hofstra/Northwell, Northwell Health at Lenox Hill Hospital, New York, New York, United States

**Keywords:** direct oral anticoagulant, atrial fibrillation, surgery, bleeding, thromboembolism

## Abstract

**Introduction** The Perioperative Anticoagulation Use for Surgery Evaluation (PAUSE) Study assessed a standardized perioperative management strategy in patients with atrial fibrillation who were taking a direct oral anticoagulant (DOAC) and required an elective surgery or procedure. The aim of this substudy is to analyze the safety of this management strategy across different patient subgroups, according to four presurgical variables: (1) DOAC type and dose, (2) surgery/procedure bleed risk, (3) patient renal function, and (4) age.

**Methods** Clinical outcomes analyzed included major bleeding (MB), arterial thromboembolism, any bleeding, and any thromboembolism. We used descriptive statistics to summarize clinical outcomes, where the frequency, proportion, and 95% confidence interval were reported. Fisher's exact tests were used for testing the null hypothesis of independence between the clinical outcome and patient characteristic, where the test *p* -values were reported.

**Results** There were 3,007 patients with atrial fibrillation requiring perioperative DOAC management. There was no significant difference in bleeding or thromboembolic outcomes according to DOAC type/dose regimen, renal function, or patient age. The rate of MB was significantly higher with high bleed risk procedures than low bleed risk procedures in apixaban-treated patients (2.9 vs. 0.59%; *p* < 0.01), but not in dabigatran-treated patients (0.88 vs. 0.91%; *p* = 1.0) or rivaroxaban-treated patients (2.9 vs. 1.3%; *p* = 0.06). The risk for thromboembolism did not differ according to surgery/procedure-related bleed risk.

**Conclusion** Our results suggest that in DOAC-treated patients who received standardized perioperative management, surgical bleed risk is an important determinant of bleeding but not thromboembolic outcomes, although this finding was not consistent across all DOACs. There were no differences in bleeding and thromboembolism according to DOAC type and dose, renal function, or age.

## Introduction

The perioperative management of patients who are receiving a direct oral anticoagulant (DOAC) for atrial fibrillation is becoming an increasingly common clinical problem. Approximately one in six patients per year receiving a DOAC long-term will be assessed for perioperative management. 1 2 This is an especially high-risk period for patients where a DOAC interruption interval that is too long may place patients at risk for thrombosis and a DOAC interruption interval that is too short may place patients at risk for bleeding. The development of these complications may be related to the timing and duration of DOAC interruption, the inherent bleeding risk of the surgery or procedure being undertaken, the use of perioperative heparin bridging, and patient-specific factors including advanced age and comorbid conditions. In clinical practice, the periprocedural management of DOACs varies widely, 3 which places many patients at risk for these complications. The Perioperative Anticoagulation Use for Surgery Evaluation (PAUSE) study was the first to prospectively assess a standardized perioperative management strategy in three cohorts of DOAC-treated patients on apixaban, dabigatran, or rivaroxaban who needed an elective surgery. 4 The study's principal finding was that in the combined study population of 3,007 patients, the 30-day perioperative rates of major bleeding (MB) were low (1.4%), as were rates of arterial thromboembolism (ATE) (0.33%). 4

Despite these encouraging findings, DOAC use involves multiple agents and dose regimens, with the potential for between-DOAC differences in pharmacologic and pharmacokinetic properties, and perioperative management involves a spectrum of surgery and procedure types. These factors, along with patient-specific factors, such as renal function and age, may affect rates of perioperative bleeding and thromboembolism. It is clinically relevant, therefore, to examine the safety of the PAUSE management strategy across different patient subgroups, according to DOAC type and dose regimen used, surgery/procedure-related bleed risk, and according to patient renal function and age.

Against this background, we undertook a prespecified subgroup analysis of the PAUSE study database. Our primary aim was to compare rates of MB and ATE according to DOAC type, DOAC dose, surgery/procedure-related bleed risk, patient renal function, and patient age. Our secondary aim was to assess any clinically important bleeding and any thromboembolism across these patient subgroups.

## Methods

### Patient Population and Perioperative Management

The PAUSE cohort study was conducted at 23 clinical sites in Canada, the United States, and Europe. The study involved DOAC-treated patients with atrial fibrillation who required anticoagulant therapy interruption for an elective surgery/procedure. Participants were ≥18 years old, were receiving apixaban, dabigatran, or rivaroxaban (edoxaban was not universally available for clinical use during PAUSE), and were scheduled for an elective (i.e., planned) surgery or procedure.

Patients were separated into three cohorts according to the DOAC used and were managed with a standardized perioperative DOAC interruption and resumption strategy based on DOAC pharmacokinetic properties, surgery/procedure-related bleed risk ( Table 1 ), estimated creatinine clearance (CrCl), and patient age. Patients were classified as undergoing a high- or low bleed risk surgery or procedure according to an empiric classification scheme that was adopted from another perioperative anticoagulant management study, the BRIDGE trial. 5 DOACs were withheld for 1 day before and after a low bleed risk surgery/procedure and for 2 days before and 2 to 3 days after a high bleed risk surgery/procedure. The exception to this management was patients on dabigatran with a CrCl of 30 to <50 mL/min in whom 1 to 2 additional days of interruption was done to account for dabigatran's primary dependence on the kidney for elimination. Low-dose low-molecular weight heparin was permitted postoperatively in patients at risk for venous thromboembolism until the DOAC was resumed.

**Table 1:** Surgery/procedure classification based on bleed risk

High bleed risk	Low bleed risk
Any surgery requiring neuraxial anesthesia • Neuraxial anesthesia/injection—epidural anesthesia/injection.	Gastrointestinal procedures • Colonoscopy, gastroscopy, sigmoidoscopy—endoscopic retrograde pancreaticocholangiography—capsule endoscopy—push enteroscopy—Barrett's esophagus ablation.
Major intracranial or neuraxial surgery • Brain cancer resection—laminectomy or neuraxial tumor resection—intracranial (subdural, epidural) bleed evacuation.	Cardiac procedures: • Permanent pacemaker implantation or battery change. • Internal cardiac defibrillator implantation or battery change—artrioventricular node ablation—coronary artery angiography (radial approach).
Major thoracic surgery • Lobectomy, pneumonectomy, esophagectomy.	Dental procedures • Tooth extraction (up to two extractions) —endodontic (root canal) procedure.
Major cardiac surgery • Coronary artery bypass—valve replacement or repair.	Skin procedures and skin biopsy
Major vascular surgery • Aortic aneurysm repair—aorto-bifemoral bypass, popliteal bypass—carotid endarterectomy.	Eye procedures and phacoemulsification (cataract)
Major abdominopelvic surgery • Hepatobiliary cancer resection—pancreatic cancer or pseudocyst resection—colorectal and gastric cancer resection—diverticular disease resection. • Inflammatory bowel disease resection—renal cancer resection—bladder cancer resection—endometrial cancer resection—ovarian cancer resection—radical prostatectomy.	
Major orthopaedic surgery • Hip arthroplasty or hip fracture repair—knee arthroplasty or tibial osteotomy—shoulder arthroplasty—metatarsal osteotomy.	
Other major cancer or reconstructive surgery • Head and neck cancer surgery—reconstructive facial, abdominal, limb surgery.	

### Clinical Outcomes

The primary clinical outcomes in PAUSE and in this subgroup analysis were MB, defined by standardized criteria for surgery-related bleeding, and ATE, defined as an ischemic stroke, transient ischemic attack, or systemic embolism. 6 The secondary clinical outcomes for this analysis were clinically important bleeding, which was a composite of MB and clinically relevant nonmajor bleeding (CRNMB), and any thromboembolism, which was a composite of ATE, myocardial infarction, deep vein thrombosis, pulmonary embolism, and catheter-associated venous or arterial embolism. These clinical outcomes were assessed from the time of the first preoperative DOAC dose interruption until 30 days after the surgery/procedure and were adjudicated by an independent committee blinded to the DOAC the patients were receiving and their perioperative management.

We assessed clinical outcomes in the following patient subgroups according to: DOAC type (apixaban, dabigatran, and rivaroxaban); DOAC dose regimen (standard- or low-dose regimen); surgery/procedure-related bleed risk (high or low bleed risk); patient renal function (CrCl <50 mL/min or ≥50 mL/min); and patient age (age < 75 years and age ≥ 75 years). Patient who were taking apixaban with CrCl <25 mL/min and those taking rivaroxaban or dabigatran with a CrCl <30 mL/min were excluded from the PAUSE study and this subgroup analysis.

### Statistical Analysis

We used descriptive statistics to summarize the clinical outcomes, where the frequency, proportion, and associated 95% confidence intervals (CIs) are reported. Fisher's exact test was used for testing the null hypothesis of independence between the clinical outcome and patient characteristic. A *p* -value <0.05 was considered as statistically significant of rejecting the null hypothesis, that is, an association between the outcome and patient characteristic.

## Results

In the PAUSE study, 3,007 patients were enrolled and included in the primary efficacy analysis, out of which 1,257 patients (41.8%) were in the apixaban cohort, 668 (22.2%) were in the dabigatran cohort, and 1,082 patients (36.0%) were in the rivaroxaban cohort. A low-dose regimen was taken by 20.0% ( *n* = 252) of apixaban-treated patients (2.5-mg twice daily), by 37.1% ( *n* = 248) of dabigatran-treated patients (110 mg twice daily), and by 16.7% ( *n* = 181) of rivaroxaban-treated patients (15 mg daily). One-third (33.2%) of all patients underwent a high bleed risk surgery/procedure. Moderately impaired renal function (CrCl: 30–50 mL/min) was seen in 18.6% ( *n* = 234) of patients on apixaban, 12.0% ( *n* = 80) of patients on dabigatran, and 14.3% ( *n* = 155) of patients on rivaroxaban. Finally, 46.3% ( *n* = 582) of patients on apixaban, 44.0% ( *n* = 294) of patients on dabigatran, and 42.9% ( *n* = 464) of patients on rivaroxaban were greater than or equal to the age of 75 years.

### DOAC Type and Dose Regimen and Risk for Bleeding and Thromboembolism

As shown in Table 2 , there was no effect of the DOAC type (apixaban, dabigatran, rivaroxaban) on the outcomes of MB, ATE, any bleeding, or any thromboembolism. Similarly, as shown in Table 3 , there was no effect of the DOAC dose regimen (apixaban 5 vs. 2.5 mg, dabigatran 150 vs. 110 mg, rivaroxaban 20 vs. 15 mg) on MB, ATE, any bleeding, or any thromboembolism.

**Table 2:** Effect of DOAC type of clinical outcomes

Clinical outcome	DOAC		
	Apixaban ( *N* = 1,257)	Dabigatran ( *N* = 668)	Rivaroxaban ( *N* = 1,082)
Major bleeding			
No. % (95% CI)	17, **1.35** (0.85–2.16)	6, **0.90** (0.41–1.95)	20, **1.85** (1.2–2.84)
*p* -Value	*p* = 0.25		
Arterial thromboembolism			
No. % (95% CI)	2, **0.16** (0.04–0.58)	4, **0.60** (0.23–1.53)	4, **0.37** (0.14–0.95)
*p* -Value	*p* = 0.25		
Any bleeding (MB and CRNMB)			
No. % (95% CI)	38, **3.02** (2.21–4.12)	19, **2.84** (1.83–4.4)	45, **4.16** (3.12–5.52)
*p* -Value	*p* = 0.22		
Any thromboembolism			
No. % (95% CI)	9, **0.72** (0.38–1.36)	7, **1.05** (0.51–2.15)	5, **0.46** (0.2–1.08)
*p* -Value	*p* = 0.34		

Abbreviations: CI, confidence interval; CRNMB, clinically relevant nonmajor bleeding; DOAC, direct oral anticoagulant; MB, major bleeding.

Note: Bold values represent the percentage of patients that experienced that clinical outcome.

**Table 3:** Effect of DOAC dose regimen on clinical outcomes

Clinical outcome	DOAC dose regimen					
	Apixaban		Dabigatran		Rivaroxaban	
	2.5 mg ( *n* = 252)	5 mg ( *n* = 1,005)	110 mg ( *n* = 248)	150 mg ( *n* = 420)	15 mg ( *n* = 181)	20 mg ( *n* = 901)
Major bleeding						
No. % (95% CI)	3, **1.2** (0.41–3.4)	14, **1.4** (0.83–2.3)	2, **0.81** (0.22–2.9)	4, **0.95** (0.37–2.4)	6, **3.3** (1.53–7.0)	14, **1.5** (0.93–2.6)
*p* -Value	*p* = 1.0		*p* = 1.0		*p* = 0.13	
Arterial thromboembolism						
No. % (95% CI)	1, **0.40** (0.07–2.2)	1, **0.10** (0.02–0.56)	1, **0.40** (0.07–2.2)	3, **0.71** (0.24–2.1)	1, **0.55** (0.1–3.1)	3, **0.33** (0.11–0.97)
*p* -Value	*p* = 0.36		*p* = 1.0		*p* = 0.52	
Any bleeding (MB and CRNMB)						
No. % (95% CI)	10, **4.0** (2.2–7.1)	28, **2.8** (1.9–4.0)	8, **3.2** (1.6–6.2)	11, **2.6** (1.5–4.6)	8, **4.4** (2.3–8.5)	37, **4.1** (3.0–5.6)
*p* -Value	*p* = 0.31		*p* = 0.64		*p* = 0.84	
Any thromboembolism						
No., % (95% CI)	3, **1.2** (0.41–3.4)	6, **0.60** (0.27–1.3)	2, **0.81** (0.22–2.9)	5, **1.2** (0.51–2.8)	1, **0.55** (0.10–3.1)	4, **0.44** (0.17–1.1)
*p* -Value	*p* = 0.39		*p* = 1.0		*p* = 1.0	

Abbreviations: CI, confidence interval; CRNMB, clinically relevant nonmajor bleeding; DOAC, direct oral anticoagulant; MB, major bleeding.

Note: Bold values represent the percentage of patients that experienced that clinical outcome.

### Surgery/Procedure Type and Risk for Bleeding and Thromboembolism

As shown in Table 4 , the incidence of MB was low overall (<2%) but higher in patients having a high bleed risk surgery/procedure. In apixaban-treated patients, the rate of MB was significantly higher in the high bleed risk group (2.9%; 95% CI: 1.7–5.1) than in the low bleed risk group (0.59%; 95% CI: 0.25–1.4) ( *p* < 0.01). In dabigatran-treated patients, the rate of MB was not significantly different between the high bleed risk group (0.88%; 95% CI: 0.24–3.1) and low bleed risk group (0.91%; 95% CI: 0.35–2.3) ( *p* = 1.0). In rivaroxaban-treated patients, the rate of MB was nominally but not significantly higher in the high bleed risk group (2.9%; 95% CI: 1.6–5.2) than in the low bleed risk group (1.3%; 95% CI: 0.67–2.4) ( *p* = 0.06). Regardless of whether patients were having a high or low bleed risk surgery/procedure, the rate of ATE was low in apixaban-treated, dabigatran-treated, and rivaroxaban-treated patients.

**Table 4:** Effect of surgery/procedure-related bleed risk on clinical outcomes

Outcome	DOAC type and surgery/procedure-related bleed risk					
	Apixaban		Dabigatran		Rivaroxaban	
	High bleed risk ( *n* = 406)	Low bleed risk ( *n* = 851)	High bleed risk ( *n* = 228)	Low bleed risk ( *n* = 440)	High bleed risk ( *n* = 373)	Low bleed risk ( *n* = 709)
Major bleeding						
No., % (95% CI)	12, **2.9** (1.7–5.1)	5, **0.59** (0.25–1.4)	2, **0.88** (0.24–3.1)	4, **0.91** (0.35–2.3)	11, **2.9** (1.6–5.2)	9, **1.3** (0.67–2.4)
*p* -Value	*p* < 0.01		*p* = 1.0		*p* = 0.06	
Arterial thromboembolism						
No. % (95% CI)	1, **0.25** (0.04–1.4)	1, **0.12** (0.02–0.66)	1, **0.44** (0.08–2.4)	3, **0.68** (0.23–2.0)	2, **0.54** (0.15–1.9)	2, **0.28** (0.08–1.0)
*p* -Value	*p* = 0.54		*p* = 1.0		*p* = 0.61	
Any bleed (MB + CRNMB)						
No. % (95% CI)	23, **5.7** (3.8–8.4)	15, **1.8** (1.1–2.9)	7, **3.1** (1.5–6.2)	12, **2.7** (1.6–4.7)	25, **6.7** (4.9–9.7)	20, **2.8** (1.8–4.3)
*p* -Value	*p* < 0.01		*p* = 0.81		*p* < 0.01	
Any thromboembolism						
No. % (95% CI)	7, **1.7** (0.84–3.5)	2, **0.24** (0.06–0.85)	3, **1.3** (0.45–3.8)	4, **0.91** (0.3–2.3)	3, **0.80** (0.27–2.3)	2, **0.28** (0.08–1.0)
*p* -Value	*p* < 0.01		*p* = 0.69		*p* = 0.35	

Abbreviations: CI, confidence interval; CRNMB, clinically relevant nonmajor bleeding; DOAC, direct oral anticoagulant; MB, major bleeding.

Note: Bold values represent the percentage of patients that experienced that clinical outcome.

The rate of any (major and clinically relevant nonmajor) bleeding, remained low overall. When classified according to bleeding risk, the high bleed risk procedures had a higher rate of any bleeding. In apixaban-treated patients, the rate of any bleeding was significantly higher in the high bleed risk group (5.7%; 95% CI: 3.8–8.4) than low bleed risk group (1.8%; 95% CI: 1.1–2.9) ( *p* < 0.01). In dabigatran-treated patients, the rate of any bleeding was not significantly different between the high bleed risk group (3.1%; 95% CI: 1.5–6.2) and low bleed risk group (2.7%; 95% CI: 1.6–4.7) ( *p* = 0.81). In rivaroxaban-treated patients, the rate of any bleeding was significantly higher in the high bleed risk group (6.7%; 95% CI: 4.9–9.7) than the low bleed risk group (2.8%; 95% CI: 1.8–4.3) ( *p* < 0.01).

Finally, the rate of any thromboembolism was infrequent. It was significantly higher in high bleed risk (1.7%; 95% CI: 0.84–3.5) than low bleed risk procedures (0.24%; 95% CI: 0.06–0.85) in apixaban-treated patients ( *p* < 0.01), and did not differ in high bleed risk (1.3%; 95% CI: 0.45–3.8) and low bleed risk procedures (0.91%; 95% CI: 0.3–2.3) in dabigatran-treated patients ( *p* = 0.69), or in high bleed risk (0.80%; 95% CI: 0.27–2.3) and low bleed risk procedures (0.28%; 95% CI: 0.08–1.0) in rivaroxaban-treated patients ( *p* = 0.35).

### Renal Function and Risk for Bleeding and Thromboembolism

As shown in Table 5 , when categorizing patients based on renal function, there was no significant difference in MB, ATE, any bleeding, or any thromboembolism according to patient renal function (CrCl 25–50 mL/min vs. CrCl > 50 mL/min for apixaban, and CrCl 30–50 mL/min vs. ≥ 50 mL/min for dabigatran and rivaroxaban). Patients were excluded from the initial PAUSE study with CrCl <25 mL/min for apixaban, and <30 mL/min for dabigatran or rivaroxaban.

**Table 5:** Effect of renal function on clinical outcomes

Outcome	DOAC cohort and creatinine clearance a					
	Apixaban		Dabigatran etexilate		Rivaroxaban	
	25–50 mL/min ( *n* = 234)	>50 mL/min ( *n* = 1023)	30–50 mL/min ( *n* = 80)	>50 mL/min ( *n* = 588)	30–50 mL/min ( *n* = 155)	>50 mL/min ( *n* = 927)
Major bleeding						
No. % (95% CI)	1, **0.43** (0.08–2.4)	16, **1.6** (0.96–2.5)	1, **1.2** (0.22–6.7)	5, **0.85** (0.36–2.0)	5, **3.2** (1.4–7.3)	15, **1.6** (0.98–2.6)
*p* -Value	*p* = 0.22		*p* = 0.54		*p* = 0.19	
Arterial thromboembolism						
No. % (95% CI)	1, **0.43** (0.08–2.4)	1, **0.10** (0.02–0.55)	0, **0.0** (0–4.6)	4, **0.68** (0.26–1.7)	1, **0.65** (0.11–3.6)	3, **0.32** (0.11–0.95)
*p* -Value	*p* = 0.34		*p* = 1.0		*p* = 0.46	
Any bleed (MB + CRNMB)						
No. % (95% CI)	6, **2.6** (1.2–5.5)	32, **3.1** (2.2–4.4)	3, **3.7** (1.3–10.4)	16, **2.7** (1.7–4.4)	6, **3.9** (1.8–8.2)	39, **4.2** (3.1–5.7)
*p* -Value	*p* = 0.83		*p* = 0.49		*p* = 1.0	
Any thromboembolism						
No., % (95% CI)	3, **1.3** (0.44–3.7)	6, **0.59** (0.27–1.3)	0, **0.0** (0–4.6)	7, **1.2** (0.58–2.4)	1, **0.65** (0.11–3.6)	4, **0.43** (0.17–1.1)
*p* -Value	*p* = 0.38		*p* = 1.0		*p* = 0.54	

Abbreviations: CI, confidence interval; CRNMB, clinically relevant nonmajor bleeding; DOAC, direct oral anticoagulant; MB, major bleeding.

a Patients were excluded from the PAUSE study with CrCl <25 mL/min for apixaban, and <30 mL/min for dabigatran or rivaroxaban.

Note: Bold values represent the percentage of patients that experienced that clinical outcome.

### Patient Age and Risk for Bleeding and Thromboembolism

As shown in Table 6 , when categorizing patients based on patient age, there was no significant difference in MB, ATE, any bleeding, or any thromboembolism according to patient age (age < 75 years and age ≥ 75 years).

**Table 6:** Effect of patient age on clinical outcomes

Outcome	DOAC cohort and patient age					
	Apixaban		Dabigatran etexilate		Rivaroxaban	
	Age < 75 y ( *n* = 675)	Age ≥ 75 y ( *n* = 582)	Age < 75 y ( *n* = 374)	Age ≥ 75 y ( *n* = 294)	Age < 75 y ( *n* = 618)	Age ≥ 75 y ( *n* = 464)
Major bleeding						
No. % (95% CI)	6, **0.89** (0.41–1.93)	11, **1.89** (1.06–3.35)	3, **0.80** (0.27–2.33)	3, **1.02** (0.35–2.96)	11, **1.78** (1–3.16)	9, **1.94** (1.02–3.64)
*p* -Value	*p* = 0.15		*p* = 1.0		*p* = 1.0	
Arterial thromboembolism						
No. % (95% CI)	1, **0.15** (0.03–0.83)	1, **0.17** (0.03–0.97)	2, **0.53** (0.15–1.93)	2, **0.68** (0.19–2.45)	3, **0.49** (0.17–1.42)	1, **0.22** (0.04–1.21)
*p* -Value	*p* = 1.0		*p* = 1.0		*p* = 0.64	
Any bleed (MB + CRNMB)						
No. % (95% CI)	15, **2.22** (1.35–3.63)	23, **3.95** (2.65–5.86)	12, **3.21** (1.84–5.52)	7, **2.38** (1.16–4.83)	23, **3.72** (2.49–5.52)	22, **4.74** (3.15–7.07)
*p-* Value	*p* = 0.10		*p* = 0.64		*p* = 0.44	
Any thromboembolism						
No. % (95% CI)	6, **0.89** (0.41–1.93)	3, **0.52** (0.18–1.5)	3, **0.80** (0.27–2.33)	4, **1.36** (0.53–3.45)	4, **0.65** (0.25–1.65)	1, **0.22** (0.04–1.21)
*p* -Value	*p* = 0.52		*p* = 0.71		*p* = 0.40	

Abbreviations: CI, confidence interval; CRNMB, clinically relevant nonmajor bleeding; DOAC, direct oral anticoagulant; MB, major bleeding.

## Discussion

This study aimed to assess the effect of a standardized perioperative DOAC management strategy on bleeding and thromboembolic outcomes when considered according to DOAC type and dose regimen, surgery/procedure bleed risk, patient renal function, and patient age. There are two main findings from this subgroup analysis of the PAUSE study. First, there was no significant difference in perioperative MB and ATE in patients who were receiving apixaban, dabigatran, or rivaroxaban and there was no significant difference in these outcomes among patients who were receiving a standard or lower dose DOAC regimen, according to patient renal function and age. Second, patients having a high bleed risk surgery/procedure appeared to be at an increased risk for MB, but this finding was not consistent across DOACs. While surgical bleed risk was found to be an important determinant of bleeding, it had minimal effect on thromboembolic outcomes. The incidence of any thromboembolism was significantly higher in high bleed risk (1.7%; 95% CI: 0.8–3.5) than low bleed risk (0.24%; 95% CI: 0.06–0.85) in apixaban-treated patients ( *p* < 0.01), but this was not the case for patients treated with dabigatran or rivaroxaban. Taken together, these findings suggest that the PAUSE perioperative management is comparable in terms of safety irrespective of the type and dose of DOAC patients were receiving and patient renal function.

It is reassuring that the PAUSE management strategy is applicable to the three DOACs and DOAC dose regimens studied. Although this analysis was prespecified, the PAUSE study was not powered to detect across-DOAC group differences in bleeding and thromboembolic outcomes. However, in a companion multivariable regression analysis of the PAUSE database, DOAC type was not an independent predictor of perioperative MB. The lack of an association between renal function and bleeding outcomes may be because patients on dabigatran with a CrCl <50 mL/min had a longer preoperative interruption interval whereas most patients taking apixaban or rivaroxaban may have had dose reductions with a CrCl <50 mL/min or lower; in either case, these adjustments would have minimized residual DOAC levels at the time of a surgery/procedure. In the aforementioned multivariable regression analysis of the PAUSE database, patient CrCl was not predictive of perioperative bleeding.

There were numerically more bleeding events in patients having a high than low bleed risk surgery/procedure, but the absolute risk for high bleed risk procedures was low: approximately 2 to 3% for MB and approximately 5 to 7% for any (major and clinically relevant nonmajor) bleeding. Rates of perioperative MB in the overall PAUSE study population are comparable or lower than those observed in other perioperative antithrombotic management trials, with overall rates of MB of 2 to 3%, 5 7 8 despite accounting for different definitions of bleeding. This finding is also in line with a recent meta-analysis of periprocedural outcomes of DOACs of the large atrial fibrillation trials, which found an overall MB rate of 2.1% with a periprocedural strategy requiring DOAC interruption. 9 This is an expected finding as, intuitively, patients undergoing high bleed risk surgery, such as cardiac, vascular, major orthopaedic or cancer surgery, are expected to have more bleeding and transfusion requirements than patients having less extensive surgery or procedures. As to whether a high bleed risk surgery or procedure confers an increased risk for bleeding, there was a nonsignificant increase in MB (odds ratio [OR] 2.7; 95% CI: 1.5–5.1) and any bleeding (OR 2.3; 95% CI: 1.6–3.4) on univariate analysis of the PAUSE database independent of other factors such as increasing age and comorbidities, but surgery/procedure bleed risk was not a significant determinant with multivariate modeling. 10 The increased risk of MB in high bleed risk procedures in our analysis was comparable to a further analysis of the BRIDGE trial, which found a nearly threefold increased risk of MB (OR 2.9, 95% CI 1.4–5.9) when comparing high versus low bleed risk procedures during interruption of chronic oral anticoagulant therapy. 11 Further study is needed, to determine if specific surgery types are more susceptible to an increase in perioperative bleeding.

We acknowledge limitations in this subgroup analysis of the PAUSE database, most important of which is that the PAUSE study was not designed to have sufficient power to assess differences in bleeding and thromboembolic outcomes between patient subgroups distinguished by DOAC used, surgery type, renal function, and patient age. However, our analysis reassuringly did not find any important signal across DOACs in the subgroups studied. Our study found that when categorizing patients based on renal function, there was no significant difference in MB, ATE, any bleeding, or any thromboembolism. However, patients were categorized to either a CrCl of >50 mL/min, which was considered normal, or a CrCl <50 mL/min, which was considered reduced. Patients with CrCl less than 25 mL/min for apixaban or less than 30 mL/min for dabigatran or rivaroxaban, were excluded from the initial study design. As more patients with end-stage renal disease start using apixaban, 12 this variable may have more importance and may require further study. In the original PAUSE study, there were few patients who received neuraxial anesthesia ( *n* = 230), for whom there is a concern about increased bleeding risk associated with an excessive residual anticoagulant level. 4 Further study is required to fully characterize this high-risk surgical procedure and determine the best way to manage these patients to minimize perioperative risk. A randomized clinical trial was considered during design of the original PAUSE study but was not adopted because no alternative strategy existed that would be suitable as a control. Instead, a prospective management study was adopted. Comparison of outcomes across DOACs was therefore limited because random allocation of DOACs was not feasible within the context of perioperative management. Finally, the event rate for ATE events was extremely low which prevented meaningful analysis and limited the power to undertake comparisons. Our findings should thus be considered exploratory and hypothesis generating. Additional studies are needed, possibly using large, population-based linked databases, to corroborate the findings in this study and to assess determinants of perioperative bleeding and thromboembolism for specific DOACs. Finally, these findings may not be applicable to patients taking edoxaban, as this DOAC was not available for clinical use during development of the PAUSE study protocols and, thus, edoxaban-treated patients were excluded from PAUSE.

To summarize, among DOAC-treated patients who received standardized perioperative management, we found no significant differences in bleeding and thromboembolic outcomes according to DOAC type, DOAC dose regimen, patient renal function, and patient age. The empiric classification of surgical bleeding risk initially defined in the BRIDGE trial appears to appropriately discriminate DOAC-related perioperative bleeding risk.

## References

[1] RE-LY Investigators Healey J S Eikelboom J Douketis J Periprocedural bleeding and thromboembolic events with dabigatran compared with warfarin: results from the Randomized Evaluation of Long-Term Anticoagulation Therapy (RE-LY) randomized trial Circulation 2012 126 03 343 348 22700854 10.1161/CIRCULATIONAHA.111.090464

[2] Zulkifly H Lip G YH Lane D A Epidemiology of atrial fibrillation Int J Clin Pract 2018 72 03 e13070 29493854 10.1111/ijcp.13070

[3] Faraoni D Samama C M Ranucci M Dietrich W Levy J H Perioperative management of patients receiving new oral anticoagulants: an international survey Clin Lab Med 2014 34 03 637 654 25168948 10.1016/j.cll.2014.06.006

[4] Douketis J D Spyropoulos A C Duncan J Perioperative management of patients with atrial fibrillation receiving a direct oral anticoagulant JAMA Intern Med 2019 179 11 1469 1478 10.1001/jamainternmed.2019.2431 PMC6686768 31380891

[5] BRIDGE Investigators Douketis J D Spyropoulos A C Kaatz S Perioperative bridging anticoagulation in patients with atrial fibrillation N Engl J Med 2015 373 09 823 833 26095867 10.1056/NEJMoa1501035 PMC4931686

[6] Douketis J D Spyropoulos A C Anderson J M The perioperative anticoagulant use for surgery evaluation (PAUSE) study for patients on a direct oral anticoagulant who need an elective surgery or procedure: design and rationale Thromb Haemost 2017 117 12 2415 2424 29212129 10.1160/TH17-08-0553

[7] MANAGE Investigators Devereaux P J Duceppe E Guyatt G Dabigatran in patients with myocardial injury after non-cardiac surgery (MANAGE): an international, randomised, placebo-controlled trial Lancet 2018 391(10137):2325 2334 29900874 10.1016/S0140-6736(18)30832-8

[8] POISE-2 Investigators Devereaux P J Mrkobrada M Sessler D I Aspirin in patients undergoing noncardiac surgery N Engl J Med 2014 370 16 1494 1503 24679062 10.1056/NEJMoa1401105

[9] Nazha B Pandya B Cohen J Periprocedural outcomes of direct oral anticoagulants versus warfarin in nonvalvular atrial fibrillation Circulation 2018 138 14 1402 1411 29794081 10.1161/CIRCULATIONAHA.117.031457

[10] Tafur A Li N Clark N Predictors of bleeding in the Perioperative Anticoagulant Use for Surgery Evaluation (PAUSE) Study Blood 2019 134 01 710 10.1161/JAHA.120.017316 PMC7792425 32969288

[11] BRIDGE Investigators Clark N P Douketis J D Hasselblad V Schulman S Kindzelski A L Ortel T L Predictors of perioperative major bleeding in patients who interrupt warfarin for an elective surgery or procedure: analysis of the BRIDGE trial Am Heart J 2018 195 108 114 29224638 10.1016/j.ahj.2017.09.015 PMC5730345

[12] Siontis K C Zhang X Eckard A Outcomes associated with apixaban use in patients with end-stage kidney disease and atrial fibrillation in the United States Circulation 2018 138 15 1519 1529 29954737 10.1161/CIRCULATIONAHA.118.035418 PMC6202193

